# Facing Climate Change: Biotechnology of Iconic Mediterranean Woody Crops

**DOI:** 10.3389/fpls.2019.00427

**Published:** 2019-04-16

**Authors:** Carlos De Ollas, Raphaël Morillón, Vasileios Fotopoulos, Jaime Puértolas, Patrick Ollitrault, Aurelio Gómez-Cadenas, Vicent Arbona

**Affiliations:** ^1^Departament de Ciències Agràries i del Medi Natural, Universitat Jaume I, Castellón de la Plana, Spain; ^2^Centre de Coopération Internationale en Recherche Agronomique pour le Développement (CIRAD), Petit-Bourg, France; ^3^Department of Agricultural Sciences, Biotechnology and Food Science, Cyprus University of Technology, Limassol, Cyprus; ^4^Lancaster Environment Centre, Lancaster University, Lancaster, United Kingdom; ^5^Centre de Coopération Internationale en Recherche Agronomique pour le Développement (CIRAD), San-Giuliano, France

**Keywords:** citrus, climate change, genomics, grapevine, metabolomics, olive tree, proteomics, systems biology

## Abstract

The Mediterranean basin is especially sensitive to the adverse outcomes of climate change and especially to variations in rainfall patterns and the incidence of extremely high temperatures. These two concurring adverse environmental conditions will surely have a detrimental effect on crop performance and productivity that will be particularly severe on woody crops such as citrus, olive and grapevine that define the backbone of traditional Mediterranean agriculture. These woody species have been traditionally selected for traits such as improved fruit yield and quality or alteration in harvesting periods, leaving out traits related to plant field performance. This is currently a crucial aspect due to the progressive and imminent effects of global climate change. Although complete genome sequence exists for sweet orange (*Citrus sinensis*) and clementine (*Citrus clementina*), olive tree (*Olea europaea*) and grapevine (*Vitis vinifera*), the development of biotechnological tools to improve stress tolerance still relies on the study of the available genetic resources including interspecific hybrids, naturally occurring (or induced) polyploids and wild relatives under field conditions. To this respect, post-genomic era studies including transcriptomics, metabolomics and proteomics provide a wide and unbiased view of plant physiology and biochemistry under adverse environmental conditions that, along with high-throughput phenotyping, could contribute to the characterization of plant genotypes exhibiting physiological and/or genetic traits that are correlated to abiotic stress tolerance. The ultimate goal of precision agriculture is to improve crop productivity, in terms of yield and quality, making a sustainable use of land and water resources under adverse environmental conditions using all available biotechnological tools and high-throughput phenotyping. This review focuses on the current state-of-the-art of biotechnological tools such as high throughput –omics and phenotyping on grapevine, citrus and olive and their contribution to plant breeding programs.

## Impact of Climate Change on the Mediterranean Area: A Focus on Traditional Agriculture and Its Economic Importance

Climate change is defined as identifiable long-term changes in the state of the climate, such as increased temperatures, atmospheric CO_2_ levels, precipitations, etc. (Korres et al., 2016). It is also important to note that although there are several natural causes behind climate change that occurred over Earth’s history, the ‘Climate Change’ concept refers to variations in climatic patterns occurring over the last century and caused by anthropogenic activities that release greenhouse gasses (e.g., CO_2_, CH_3_, NO_2_, etc.). Studies on climate change over the last two decades have rendered consistent projections that predicted a net increase of average temperatures and a significant variation in the patterns of precipitation (Lobell and Gourdji, 2012). Despite changes in the climatic patterns differ among world regions increasing evidence points that the Mediterranean area will be one of the most sensitive to the effects of climate change, the primary effects of which will be a decrease in rainfall and a sharp increase in temperatures (IPCC, 2014; Korres et al., 2016).

The increase in summer temperatures is projected to be very large in south-western parts of Europe (exceeding 6°C in some parts of France and the Iberian Peninsula) by the end of the 21st century. Annual rainfall is expected to decrease by 10% in this area compared with values recorded in the period comprising years 1961–1990 (Olesen et al., 2011).

These adverse conditions will influence productivity by affecting overall plant performance but also influencing phenology (especially relevant to perennial crops). To this respect, alteration of the climatic patterns leading to an extended growth season with no quiescence period (cold season) could dramatically reduce productivity and/or quality of the production (Gordo and Sanz, 2010). Nevertheless, not all changes in the environmental conditions pose a threat, an example being increased atmospheric CO_2_ fertilization and the extension of growing season which could improve yields in some species or circumstances (Kimball, 2016).

**FIGURE 1:**
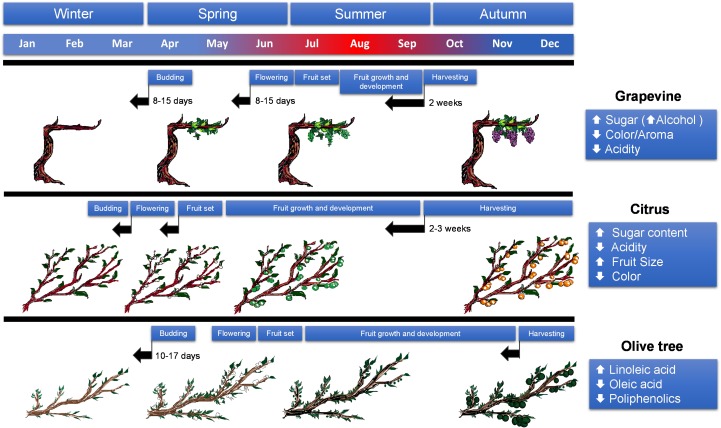
Summary of changes in phenology and fruit quality in grapevine, citrus and olive in response to drought and high temperatures derived from climate change conditions. Black arrows indicate advances in the phenological state, blue–red coloring in the month scale indicates incidence of high (red) and low (blue) temperatures.

Regarding the effects of increasing average temperatures on perennial cropping systems, it is important to note that the severity of the effects will depend on the phenological state: (a) in winter, these increases will affect early phenological events such as flower bud induction, (b) in spring, elevated temperatures could affect the persistence of already developed flowers and (c) during the fruit development phase, comprising fruit expansion and maturation, higher temperatures coupled to extreme isolated events could affect final yield and quality as well as plant performance (Figure 1).

Changes in atmospheric CO_2_ concentrations will be accompanied by severe water limitation and increases in average temperatures, posing several stress combinations with different outcomes (Zandalinas et al., 2017). For instance, Medlyn (2011) showed that stomatal conductance and transpiration in woody plant species could decrease up to a 21% in parallel to CO_2_ concentration rise, implying an increase in water use efficiency and subsequently an improvement of plant performance under water stress conditions. However, a concomitant increase of ambient temperature will increase evapotranspiration. Under these circumstances, the potential gain in yield caused by the higher CO_2_ levels (Vara Prasad et al., 2005) would be compensated and even turned in net losses due to the negative effect of high temperature once the optimum values are surpassed (Fares et al., 2017; Zandalinas et al., 2017). Therefore, the identification of traits associated to greater yields under high ambient CO_2_ constitute a desirable breeding target in order to compensate losses due to episodes of extremely high temperatures (Bunce, 2017).

The influence of changes in CO_2_ and temperature on plant performance and/or productivity is genotype-dependent. Comparing responses of rice, soybean and citrus to combined high CO_2_ and temperature, (Baker and Allen, 1993) showed that citrus displayed the greatest increase in growth due to enhanced water use efficiency and photosynthetic rate associated to CO_2_ enrichment. More recently, Vu et al. (2002) showed that ‘ambersweet’ orange trees grown at high CO_2_ (720 ppm) and temperature (up to 6°C above ambient) showed improved overall photosynthetic rate suggesting a good acclimation capacity of citrus to climate change conditions. In grapevine, FACE experiments (Free Air CO_2_ Enrichment) had shown that increased CO_2_ may stimulate wine grape production without causing negative repercussions in table grape or wine quality (Bindi et al., 2001; Moutinho-Pereira et al., 2009). Similarly to citrus, the combination of high temperature and CO_2_ had no deleterious effect in grapevine yield (Kizildeniz et al., 2018), contrary to water stress that significantly affected yield when applied along with elevated temperature. The available reports on olive trees indicate a similar behavior to atmosphere CO_2_ enrichment (Tognetti et al., 2002).

Crop productivity in Southern Europe is already limited, mainly due to water scarcity and elevated temperatures. Taking as an example the heat wave that occurred in 2003, with temperature rises of 3–5°C and annual precipitation deficit of 300 mm that led to a decrease in gross agricultural production over a 30% in Europe, it is likely that climatic conditions similar to those of the year 2003 will be more frequent in the future (Samaniego et al., 2018).

Besides the decrease in productivity and plant performance, alterations in fruit quality will also impact farmers’ income and competitiveness. Harvesting periods of certain fruit varieties (e.g., citrus) are established in relation to their optimal ripening and so are the farmers’ efforts and distribution networks. This is likely to be dramatically altered by climate change conditions (Fitchett et al., 2014). To this respect, alteration in the climatic patterns, defined by the alternation in cold and warm periods, has already had a significant effect on flowering and ripening of several perennial species (Figure 1), in some cases occurring 2 or 3 weeks earlier than in previous cropping seasons (Fitchett et al., 2014).

This review focuses on three major woody crops from the Mediterranean area: grapevine, olive and citrus. Despite having diverse botanical origins, their impact on Mediterranean region has shaped economy and culture for centuries. These *iconic* fruit crops face the challenge of remaining productive delivering the highest quality in a climate change scenario. This can be achieved following two strategies: one aimed at optimizing the available natural resources and the other at providing novel plant material better adapted to unfavorable conditions.

(a) Irrigation management, which aims at improving the efficiency or the volume of water used by the plant in relation with the total amount applied, and also water productivity, the ratio between crop yield and water used, which is normally increased by applying less water than the potential evapotranspiration demand of the crop. However, this reduction needs to be carefully applied to maintain yield (Fereres and Soriano, 2007). For Mediterranean woody crops, deficit irrigation not only enhances water productivity as it decreases transpiration, but also improves fruit quality and reduces excessive vegetative growth (Fernández et al., 2018).
(b) Breeding programs for drought-tolerant cultivars. The recent advances in phenotyping and genomics offer the possibility to find new cultivars that can achieve acceptable yields with less water. However, improvement in tolerance to water deficit is particularly elusive due to the complexity of plant responses to drought, in particular concerning with grain and fruit yield. A correct definition of the drought scenario and the crop species physiology under drought is crucial for an efficient selection (Tardieu et al., 2011). Once determined the key processes conferring drought tolerance for a certain species in the target drought scenario, the genes associated to those processes can be identified to assist in breeding. This highlights the role of crop ecophysiology in modern breeding for drought tolerance, which is often neglected (Lawlor, 2013).

In the following lines, the state-of-the-art of the application of biotechnological tools to boost up breeding programs to produce more resilient plant material will be reviewed and discussed.

## Grapevine, Citrus and Olive, Traditional Mediterranean Woody Crop Species

### Grapevine

The grapevine (*Vitis vinifera*) belongs to the family Vitaceae. The *Vitis* genus includes about 60 species distributed in Europe, North America, and Asia under temperate Mediterranean and subtropical climatic conditions. *Vitis* is separated in two subgenera, *Euvitis* and *Muscadinia* characterized by different chromosome number (2*n* = 38 and 2*n* = 40, respectively). Sexual compatibility is wide within each subgenus but most of the inter-sub-generic hybrids are sterile. Nearly all grape cultivars produced for fruit, juice and wine are classified as *Vitis vinifera* L. subsp. *vinifera* (or *sativa*) or are hybrids of *V. vinifera*. Current knowledge points toward its domestication from the wild relative of the same species: *Vitis vinifera* L. subsp. *sylvestris* (Gmelin) Hegi (This et al., 2006; Mihaljević et al., 2013). Other *Euvitis* species such as *V. rupestris*, *V. riparia* or *V. berlandieri* exhibit significant resistance to Phylloxera, Oidium, and mildews and are used primarily for rootstock breeding. It is believed that the domestication of grapevine originated in a region located between Black Sea and Iran 6,000–9,000 years ago (Myles et al., 2011). From this spot, it spread toward Middle East and Central Europe which are nowadays considered secondary domestication centers resulting from gene flow between wild and cultivated gene pools (Arroyo-Garcia et al., 2006; Myles et al., 2011; Bouby et al., 2013). In the Mediterranean Basin, grape spread gradually westwards (Terral et al., 2010), with the most ancient evidence of viticulture found in Greece and Crete (fifth millennium BC), then in Italy (ninth century BC) and Spain (early within the last millennium BC). The French viticulture appears to be more recent (600 years BC).

Winegrapes are among the most profitable crops with a global market value of 30 billion^1^ €. The most widespread winegrape varieties are: Cabernet Sauvignon, Chardonnay and Pinot Noir that are clonally propagated subsequently showing little genetic diversity among them. Winemakers are bound to deliver a very specific product and changes in climatic conditions make necessary to adjust management practices, variety or even the clone within a variety, to satisfy consumer demands (Van Leeuwen et al., 2004). Aside from yield, final grape biochemical composition, in terms of metabolite concentration, largely depends on climatic conditions, as summarized in Kuhn et al. (2014).

Winegrape phenology is also very sensitive to climate change. Indeed, the timing of phenological events has changed between 1 and 2 weeks over the past decades (Wolkovich et al., 2017 and references therein). Regulation of timing of phenological events is a complex trait influenced by climate (determined by a subset of environmental cues), genetics, and a complex interaction of both factors. However, climate and more specifically temperature is the dominant factor controlling the pace of phenological events (de Cortázar-Atauri et al., 2009) within each cultivar (Wolkovich et al., 2017). The main phenological events linked to overall production and quality in grapes are (i) budburst, that requires daytime temperatures above 10°C to initiate growth, (ii) flowering that is generally accelerated by high temperatures, (iii) veraison (or color break) followed by (iv) ripening, these two being positively regulated by high temperatures although excessive exposure to high temperatures can be detrimental, and, finally, (v) maturity, that is determined by sugar accumulation, for which temperature is critical setting the optimal harvest date (Figure 1). Martínez-Lüscher et al. (2016) evaluated the sensitivity of grapevine at different phenological stages to environmental conditions associated to climate change. According to their results, thermal time models seem to predict very accurately early phenological phases (budburst and flowering), but, as the growing season advances, other factors such as different cultural practices and water availability influence the timing of phenophases. According to Ferrise et al. (2013), the sequence of developmental processes will be accelerated advancing phenological phases, such as budbreak, flowering or ripening, with values ranging from 8 to 15 days. In general, the reproductive phase was the most affected resulting in shorter periods by an average of 6 days.

### Citrus

Citrus belong to the Rutaceae family, Aurantioideae subfamily. Most of the cultivated citrus species are part of the *Citrus* genus (Tanaka, 1961; Swingle and Reece, 1967). Molecular studies (Nicolosi et al., 2000; Barkley et al., 2006; Garcia-Lor et al., 2013; Curk et al., 2015, 2016) and recent genomic studies (Curk et al., 2014; Oueslati et al., 2017; Wu et al., 2018) provided a clear understanding of the evolution of cultivated *Citrus*, revealing the existence of four ancestral taxa: [*Citrus maxima* (Burm.) Merr. – the pummelos, *Citrus medica* L. – the citrons, *Citrus reticulata* Blanco – the mandarins and *Citrus micrantha* Wester a wild papeda species]. The other cultivated species [*Citrus aurantium* L. – sour orange, *Citrus sinensis* (L.) Osbeck – sweet orange, *Citrus paradisi* Macf. – grapefruit, *Citrus limon* (L.) Burm. F. – lemon and *Citrus aurantifolia* (Christm.) Swingle – lime] resulted from recombination among ancestral taxa. Several genera (*Poncirus*, *Fortunella*, *Eremocitrus*, *Microcitrus*, and *Clymenia*) are sexually compatible with *Citrus* species and constitute the true citrus group (Swingle and Reece, 1967). They have interest as parentals for rootstock breeding due to their tolerance traits to several abiotic stresses and diseases. Citrus species were domesticated in Southeast Asia several thousand years ago. The first species known in the Mediterranean was Citron, probably introduced by Alexander the Great to Persia and Greece from India. Old mosaics show that the Romans may have known lemon fruits around 100 years BC. Citrus were then spread throughout Europe and North Africa during the expansion of the Arabic empire. Citron, sour orange, lemon, lime, and pummelo are described in tenth and eleventh century books from Spain. Modern types of sweet oranges were introduced in Europe by the Portuguese in the early sixteenth century. The introduction of mandarins is more recent (early nineteenth century). The Mediterranean Basin is considered a secondary area of Citrus diversification, particularly important for sweet oranges and lemons (Aubert, 2001).

Irrigation requirements for Citrus production in the Mediterranean area are relatively high (Carr, 2012). In this sense, water management in the last decades has improved partly as a result of the implementation of drip irrigation. Compared with traditional surface irrigation techniques, drip irrigation allows a more accurate control of watering and optimizes water usage without a significant decrease in production. Moreover, drip irrigation facilitates management of spontaneous weeds and optimal fertilization (Carr, 2012).

At present, Mediterranean citrus fruit production and commercialization is focused on delivering the maximum fresh fruit quality, including external appearance (such as shape, texture, and color) and taste characteristics (sugar content, acidity etc.) as the main quality traits that must fit stringent parameters to successfully reach the market. To accomplish this goal, citrus production has been subjected to extensive technification including the accurate selection of genotypes to be used as rootstocks, providing adequate yield and plant performance traits to the grafted variety.

A warmer climate will affect fruit metabolism in terms of yield but also influence internal quality in several ways. High average temperatures increase sugar content and reduce acidity. This is probably linked to enhanced photosynthetic and fruit sink activities along with citric acid metabolism (Albrigo, 2004; Cercós et al., 2006). The ratio between total titrable acidity (TTA) and total soluble solids (TSS) is the main parameter used to determine optimal fruit maturity for harvesting. Hence, the adequate balance between sugars and citric acid, confers the characteristic flavor of citrus in temperate world regions in clear contrast to those produced in tropical areas (Albrigo, 2004). Moreover, external fruit quality is also affected in tropical climates increasing the occurrence of bigger fruits with smoother surfaces and lower rind color intensity (Reuther and Rios-Castaño, 1969). The development of an optimal external quality requires cooler fall/winter temperatures, whereas optimal pulp and juice quality is usually associated to temperate climates with warmer winters and higher relative humidity (Reuther, 1973).

Final fruit yield and quality in citrus depend on a series of concatenated processes, flowering, fruit set and fruit growth and development that are particularly affected by climate change conditions. Sprouting in citrus is independent of the air temperature so it can happen at any moment of the year if soil temperature is above 12°C. Under typical Mediterranean conditions, there are three sprouting events during the year (spring, summer, and fall). Spring flushes generate flower and fruit-bearing branches after flower bud induction during winter (Garcia-Luis et al., 1995). Flower bud induction is linked to environmental factors such as drought and cool temperatures (below 20°C), this latter being the primary factor. In citrus, the key phenological events in determining final fruit yield are bud floral transition and initial fruit set, that take place during winter and spring in temperate climates. Most cultivated citrus varieties are parthenocarpic or autoincompatible, therefore requiring pollination to initiate ovary-fruit transition but without fertilization of ovules (Talon et al., 1992). Climate change models predict average temperature rises between 2.6 and 4°C by the end of the 21st century, that will likely delay flowering and reduce flower number (Albrigo, 2004). Increases in winter average temperatures will definitely affect flower bud induction and reduce the number of reproductive branches on spring. As a result, number of flowers could be drastically reduced, increasing the chances of suffering heat stress and massive flower drop (Albrigo and Saúco, 2004).

The effect of high temperatures during fruit set, which has a direct influence on final production and quality, has not been extensively studied. Nevertheless, higher fruit drop rates are an expectable consequence of elevated temperatures during May–June. Fruit set is regulated by a series of factors (number of flowers, competition between reproductive organs, plant hormones, and crop load) that are, to some extent, manageable. Efficient fruit set appears to be a result of a complex interplay of several plant hormones (Iglesias et al., 2007), the activity of which can be mimicked by exogenous treatments. On the other hand, environmental factors, such as high temperatures that increase evaporative demand, cannot be controlled and need to be overcome by the available means (Iglesias et al., 2007).

Changes in phenology derived from climate change are possibly the most important factor affecting citrus production in terms of marketability and profitability. Variations in the climatic patterns of citrus-producing areas will drastically influence tree phenology, flower and fruit development as well as its organoleptic composition (Figure 1). Final fruit size is primarily determined during phase II of fruit development and it is associated to cell elongation, it is inversely correlated with number of competing fruitlets and also air temperature, as it regulates transpiration and, hence, water absorption. Optimal temperatures for enhancement of fruit growth rate appear to be in the 20–25°C range, temperatures outside this range will cause a reduction in fruit size either by slowing down growth rate or by increasing competition between developing fruitlets (Reuther, 1973). To this respect, soil moisture is also important to fruit growth rate throughout all stages of fruit development but particularly during early development. Flowering and fruit expansion characteristics need to be understood in relationship to fruit maturation and anticipated quality to maximize quality in subtropical-to-tropical climates.

### Olive

Olive (*O. europaea* L.) is part of the Oleaceae family. The *Olea* genus includes 33 species and 9 subspecies (Green, 2002), six being in the *O. europaea* species and displaying specific geographical distributions: *Olea europaea* subsp. *europaea* (Mediterranean Basin), *O. europaea* subsp. *cuspidata* (from South Africa to South Asia), *O. europaea* subsp. *laperrinei* (Saharan mountains), *O. europaea* subsp. *maroccana*, *O. europaea* subsp. *cerasiformis*, and *O. europaea* subsp. *guanchica* (Macaronesia). Cultivated and wild relative Mediterranean olive species are relatively defined as *O. europaea* subsp. *europaea* var. *europea* and *O. europaea* subsp. *europaea* var. *sylvestris*. Archaeobotanical and molecular studies suggested that domestication occurred five to six thousand years ago in the Middle East (Besnard et al., 2018). Secondary diversification of the crop followed the oleiculture diffusion over the whole Mediterranean basin (Besnard et al., 2001). However, whether multiple or single domestication events occurred is still under debate (Diez et al., 2015).

Propagation of olive trees has been traditionally carried out vegetatively by cuttings, although grafting of elite varieties on appropriate rootstocks is nowadays attracting interest as a tool to develop new groves with high density plantations. Indeed, grafting of well-established and -adapted cultivars on dwarfing rootstocks appears a good alternative to develop new cultivars suitable for high-density planting (Rugini et al., 2016). Unfortunately, the availability of such rootstocks is very limited and only a few accessions are currently under investigation (Rugini et al., 2016).

Olive trees have been domesticated for the past six millennia (Kaniewski et al., 2012), and olive oil, together with wine, are typical agricultural products linked to historic Mediterranean civilizations. Although traditional olive tree cultivation was carried out in sparse rain-fed tree plantations with 100–300 trees per hectare, in the last two decades modern intensification techniques are quickly changing the aspect of orchards, which are now planted at high density, with intensive irrigation and fertilization management (Tous et al., 2008). While improvements in cultivation techniques have led to large increases in olive oil production, prices are decreasing and, since input costs are steadily growing, the profitability of many olive plantations is at risk (Rodriguez Cohard et al., 2017). Climate change might exacerbate these issues as it is expected to reduce yields by 3.5–7% (Ronchail et al., 2014).

Unlike other crops cultivated in the area, olive trees originated and diversified in the Mediterranean basin (Besnard et al., 2018) subjected to its typical climate characterized by hot and dry summers and high inter-annual rainfall variability. Therefore, physiological and phenological characteristics are well suited to this type of climate (Connor, 2005). Moreover, despite of this restricted geographical origin, it has been suggested that several independent, disperse and widely spread domestication events could have led to the selection of locally adapted genotypes and the existence of a high number of olive varieties (Lavee and Zohary, 2011).

As in other fruit crops, reproductive development is one of the main agronomical features that will be affected by climate change. Climate conditions before and during flowering and fruiting are of pivotal importance, first determining the number of flowers, initial fruit set and, subsequently, fruit yield. In olive trees, temperature and rainfall between autumn and spring (pre-flowering and beginning of flowering periods) determine the abundance and phenology of flowering (Aguilera and Valenzuela, 2012). Different flowering models coupled with climate predictions show that by the end of the 21st century, flowering could be anticipated by 10–17 days due to increasing temperatures during the pre-flowering period (Aguilera et al., 2015; Gabaldon-Leal et al., 2017). This could be advantageous, since shifting the critical flowering time frame from the warmer to the cooler periods would reduce heat-induced flower drop and, hence, increase the fruitlet success rate. However, within the current distribution area of olive tree orchards this might not be enough to cope with increasing temperatures during flowering in the southernmost locations, as the expected rise in spring temperatures and especially during flowering can have a deleterious impact on olive production (Gabaldon-Leal et al., 2017). Olive trees produce two types of inflorescences: perfect or hermaphrodite and imperfect or staminate, containing only a residual atrophied pistil. In a recent work, a 4°C temperature rise caused a reduction in the percentage of perfect flowers and fertile inflorescences as well as fruit set (Benlloch-González et al., 2018). Flowering seems to be controlled by both genetic and environmental factors (Moreno-Alias et al., 2012). To this respect, selection of early-flowering varieties could constitute an alternative to adapt olive production to climate change in particularly sensitive areas.

Water scarcity due to rainfall reduction is the other main threat to olive production. Water stress not only has a negative impact on flowering but also reduces stomatal aperture and carbon assimilation (Hernandez-Santana et al., 2017). At present, intensification is rapidly shifting the cultivation method from extensive rain-fed groves to irrigated orchards; therefore, the forecasted reduction in water availability will require the generalization of water-saving irrigation techniques. To this respect, regulated deficit irrigation can save considerable amount of water in high-density orchards without significant yield penalty (Gomez-del-Campo, 2013). Hence, alternating partial root-zone drying, in which a reduced amount of water is applied to half of the root system, switching sides periodically, can increase notably water use efficiency in olive trees, being alternation timing a key factor in determining the results (Wahbi et al., 2005). The implementation of new, more precise, irrigation scheduling tools as well as the generation of new knowledge on physiological responses to water deficits might contribute to improve the efficiency of this strategy (Fernández et al., 2018).

Olive tree actually constitutes an interesting model woody crop species to study physiological responses to drought under Mediterranean climate for several reasons: (1) the domestication of olive tree was carried out under Mediterranean conditions, where it adapted remarkably to cope with soil and air water deficits, combining an effective control of water losses with high tolerance to desiccation (Díaz-Espejo et al., 2018), (2) it constitutes an important income crop in Southern Europe (comprising countries such as Spain, Italy, Greece, etc.); therefore, a great deal of information on stress responses at the plant performance and productive levels is available (Fernández, 2014), (3) its genome has already been sequenced (Unver et al., 2017). This information can be used to design effective tools for irrigation scheduling based on tree physiology (Aguero Alcaras et al., 2016; Aissaoui et al., 2016; Hernandez-Santana et al., 2016) and attain genotype selection based on genetic makers (Sebastiani and Busconi, 2017). However, despite the abundant information existing on drought tolerance traits among different cultivars (Guerfel et al., 2009; Trentacoste et al., 2018), crop species with long life cycles pose difficulties for breeding. This, along with the high adaptation of the species to the local climate, has probably discouraged breeding for drought tolerance in this species.

### Plant-Pollinator Interaction Under Climate Change Conditions

Climate change affects plant and animal phenology, their interaction, demography as well as their distribution in natural habitats. To this respect, the effect of global warming on the plant-pollinator interaction through variations in their phenology and range (Morton and Rafferty, 2017) is of special relevance in agronomy. As mentioned above, pollination is of crucial importance in determining fruit yield in grapevine and olive (Benlloch-González et al., 2018) and, to a lesser extent, in citrus, particularly in autoincompatible genotypes such as mandarins (Talon et al., 1992). Unfortunately, this has been poorly investigated in woody crop species, but in ephemeral spring weeds, a clear de-synchronization has been observed between flowering and the incidence of their natural pollinators, bumble-bees, leading to a phenological mismatch and a reduction in reproductive success (Kudo and Ida, 2013). In addition to potential phenological mismatches between crop plants and pollinators, changes in pollinator-flower preferences are likely to occur due to increasing ambient temperature. Several studies have shown that bees prefer to collect warm nectar from flowers at low ambient temperatures, but when air temperature rises above 30°C, they usually switch their preferences to cooler flowers (Shrestha et al., 2018). For all these reasons, it is important not to disregard the effects of climate change on insect pollinators: their population trends and phenology in the equation, besides the direct effects on woody crops.

## Selection of Improved Cultivars

Plant breeding is an important way to improve crop adaptation to abiotic stresses in the context of global climate change. At the commercial level, all three woody crops are propagated vegetatively. Therefore, any elite genotype can be clonally multiplied irrespective of the complexity of its genomic structure. For a long time, this clonal selection was based on natural bud sprouts identified in the field. Today, many modern breeding projects rely on sexual reproduction to exploit favorable traits identified in germplasm accessions, but these initiatives are hampered by the extended juvenile phase existing in most woody perennial plants (Warschefsky et al., 2016). Olive trees present the longest juvenile period of all three species, requiring more than 12 years after seed germination to induce flowering (Santos-Antunes et al., 2005). Grapevine displays the shorter juvenile phase, up to 3 years, which is morphologically characterized by the absence of tendrils in the first 9–15 nodes (Mullins et al., 1989). Most cultivated citrus species exhibit an intermediate behavior, requiring 4–8 years to start flowering, depending on the genotype and environmental conditions (Ollitrault and Navarro, 2012). In most fruit woody crops, rootstock selection constitutes an essential component in pest and disease resistance and also for plant adaptation to various abiotic stresses and particularly water deficit, soil quality, or cold (Warschefsky et al., 2016). Rootstock breeding offers the possibility to expand the selection objectives and criteria in order to adapt elite cultivars to different environmental and soil conditions.

### Breeding Objectives and Traditional Breeding Methods

#### Grapevine

Hybridization between Euvitis species is efficient, opening the way for a large germplasm exploitation. The number of *V. vinifera* cultivars is estimated to be close to 5,000 and large germplasm collections are managed in most grape-growing countries (Reisch et al., 2012). Some breeding objectives are common for wine and table grapes, concerning mainly disease resistances and adaptation to organic and sustainable viticulture, for which hybrids within *V. labrusca* germplasm collection are used. Although not very widespread among grapevine-producing regions, adaptation to very cold temperatures (-20 to -35°C) is key in certain cultivation areas and, hence, considered in their respective breeding programs. For wine grape, the important traits are (a) the adaptation to vinification, and (b) aroma and flavors since these traits can be unfavorably affected when using other species than *V. vinifera* for breeding. For table grapes, the absence of seeds in berries is an important trait as well as the extension of the harvesting period. Rootstock breeding constitutes also an essential aspect in grapevine management and production. Adequate rootstock selection allows transferring resistance traits to the grafted variety such as soil-borne pests and diseases (including the damaging Phylloxera), nematodes, etc. In addition, rootstock provides several vigor and developmental advantages. Moreover, adaptation of plants to varying soil conditions and other abiotic constraints predominant in the Mediterranean region is also mediated by rootstock (Pavlousek, 2011). As an important constraint for agriculture, significant progress has been made toward the identification of phenotypical traits associated with water stress tolerance of grapevine rootstocks (Marguerit et al., 2012; Barrios-Masias et al., 2015). Indeed, several studies demonstrated the positive role of drought-tolerant rootstocks on the control of the scion leaf stomatal conductance and canopy transpiration (Serra et al., 2014).

#### Citrus

The agro-morphologic variability of citrus is very large. Several sources of tolerance to abiotic stresses have been identified (Krueger and Navarro, 2007): tolerance to salinity of Rangpur lime (*Citrus limonia* Osb.), *Citrus macrophylla* Wester and Cleopatra mandarin (*Citrus reshni* Hort. Ex Tan.); tolerance to water deficit of Rangpur lime (*C. limonia* Osbeck) and the *Microcitrus* and *Eremocitrus* species; tolerance to iron chlorosis of Rough lemon (*Citrus jambhiri* Lush), *C. macrophylla* Wester, Volkamer lemon (*C. limonia* Osbeck), and *C. amblycarpa* (Hassk.) Ochse, cold tolerance of satsuma mandarins (*Citrus unshiu* Marc.), Kumquats (*Fortunella* sp.) and trifoliate orange, *Poncirus trifoliata* (L.) Raf. This variability opens very broad prospects for the exploitation of citrus genetic resources for adaptation breeding particularly at the rootstock level (Cimen and Yesiloglu, 2016). The facultative apomixes of many citrus species allow clonal rootstock propagation by seed and contributed to the generalization of grafting in the citrus industry. Sexual breeding programs carried out in Florida by the end of the 19th century provided several intergeneric (*Citrus* × *Poncirus*) hybrids, still used nowadays as rootstocks in many countries. These include some citrumelos (*C. paradisi* × *P. trifoliata* cvs. Swingle, Sacaton and 4475) and, particularly, the citranges (*C. sinensis* × *P. trifoliata* cvs. Troyer, Carrizo and C-35). However, most of them present some susceptibility to adverse abiotic conditions (alkalinity, salt), urging the development of a new range of intergeneric (*Citrus × Poncirus*) hybrids. Crosses between mandarins and trifoliate orange appear very promising to combine tolerances to abiotic and biotic constraints both by sexual breeding or somatic hybridization (Dambier et al., 2011).

#### Olive

Due to the high success of the selected varieties in the local climate and under the traditional extensive rain-fed cultivation techniques, genotypic selection has not been as efficient as in the rest of woody crops reviewed here, and most of the new cultivars recently released are clones of traditional varieties (Lavee, 2013). However, the challenges posed by climate change and, in particular, the new intensive methods of cultivation, urge the introduction of new varieties better adapted to the coming climate conditions. For instance, traditional varieties with low vegetative vigor seem to be better suited for high-density cultivation in hedgerows (Tous et al., 2008), opening the possibility to use these genotypes for breeding of new varieties and rootstocks adapted to this type of cultivation (Rugini et al., 2016). Diploid and tetraploid dwarfing rootstocks have been used to reduce total plant height as well as maintaining oil quality, making them interesting candidates for intensive cultivation. Olive tree germplasm resources encompass more than 1,500 cultivars with wide genetic diversity that could be useful for conventional breeding to improve stress tolerance (Baldoni and Belaj, 2010). The most amenable wild germplasm resource to exploit is *O. europaea* subsp. *europaea* (Klepo et al., 2013) bearing several abiotic stress tolerance traits (Romero-Aranda et al., 1997; Mulas, 1999; Baldoni et al., 2006), as well as improved resistance to several pests and diseases (Ciccarese et al., 2002; Mkize et al., 2008). However, the use of wild germplasm resources for olive tree breeding is still very limited (Besnard et al., 2001; Caceres et al., 2015). At present, the main breeding objectives are to select new cultivars and rootstocks with adaptation to different adverse environmental conditions and to implement modern farming practices in order to meet consumers’ demands for oil production and olives for fresh consumption (Medina et al., 2012; Diez et al., 2015). The Gene Pool Method (GPM) was recently applied to olive tree breeding in order to integrate a broader diversity; it is based on information from phylogeny, domestication, and hybridization affinity among cultivated, wild ecotypes, and other *Olea* species (Rugini et al., 2016).

### Bridging Genotype and Phenotype; Toward Marker Assisted Selection (MAS) and Genomic Selection (GS)

Recent advances in genomics are expected to greatly improve the efficiency of plant breeding for the intensification of agriculture under environmental constraints, thus increasing resilience of crops to climate change (Morrell et al., 2011; Abberton et al., 2015; Scheben et al., 2016). It will be particularly useful for efficient exploitation of adaptation traits present in germplasm accessions (Huang and Han, 2014) and to expand the gene pool for crop improvement (Brozynska et al., 2016). For the three woody crop species included in this review: grapevine, citrus and olive, rich germplasm collections exist, including commercial varieties and wild relatives (Arroyo-Garcia et al., 2006; Krueger and Navarro, 2007; Sebastiani and Busconi, 2017). Moreover, these three species have already been sequenced, further expanding the possibilities for the identification of candidate tolerance genes and contributing to bridge genotype and phenotype for adaptation traits.

The identification of molecular markers linked to tolerance to climate change becomes essential to efficiently drive breeding projects targeting Mediterranean crops. Quantitative Trait Loci or QTLs analysis and genetic association studies are based on genetic linkage (low recombination rate) of marker genes to genes directly involved in phenotype diversity. In contrast to QTL, analysis in sexual recombining populations, genome-wide association studies (GWAS) allow exploring linkage associations in more diverse germplasms and the identification of key genes that should be monomorphic in a single hybrid population. It also poses the advantage of bypassing the construction of designed segregating populations saving time and work, constituting a serious alternative in perennial fruit crop breeding programs where breeding materials are derived from many parental combinations. As a result, the usage of GWAS in fruit crops increased during the last 2 years (Iwata et al., 2016; Farneti et al., 2017; Minamikawa et al., 2017). However, its applicability depends greatly on the genetic structure of the considered germplasm and particularly the decay of the linkage disequilibrium (LD) with the genetic distance. The LD decay can be strongly affected by the evolution history of the gene pool as observed in Citrus (Garcia-Lor et al., 2012). Genomic selection (GS) is a more recent approach, initially developed for animal breeding to make an improved use high throughput molecular in marker assisted selection. It is based on modelization of the phenotypic value from high density marker information over the whole genome (Desta and Ortiz, 2014). Previous studies on animals and plants highlighted the interest of GS, especially for capturing small-effect quantitative trait loci. GS is now routinely applied in animal breeding (Meuwissen et al., 2016). Its application resulted in increased genetic gain in dairy cattle breeding where the reliability of genomic prediction exceeds 0.8 for production traits and 0.7 for fertility and other traits (Lund et al., 2011; Wiggans et al., 2011). In Canada, for example, the rate of genetic gain has approximately doubled since GS was introduced. In plants, promising results were obtained, particularly in cereals with genetic gain of GS higher than that of MAS or conventional pedigree breeding (Wang X. et al., 2018). The combined analysis of multiple traits and/or multiple environments will be important for improving the accuracy of prediction and to promote the use of GS in plant breeding projects.

QTL studies, GWAS, and GS advances can be hampered by the current knowledge on genomics and genetic marker resources development. The application of Next Generation Sequencing (NGS) to reduced genome representation, with methods such as restriction-site associated DNA sequencing (RADseq; Miller et al., 2007) or genotyping by sequencing (GBS; Elshire et al., 2011) is particularly promising. These methods allow deep coverage of the regions adjacent to restriction sites and offer great potential for high-throughput genotyping of entire genomes and for Single Nucleotide Polymorphism (SNP) mining at the same time. They are therefore very well suited for the analysis of large segregating progenies and marker trait association studies based on LD (Baxter et al., 2011; İpek et al., 2016; Montero-Pau et al., 2017). Compared with microarray analyses, these methods present the advantages of flexibility and the lack of requirement to refer to a set of pre-determined polymorphisms. High-throughput genotyping at affordable costs opens the way to GS that is much more efficient than marker-assisted selection (MAS) to increase genetic gain of complex traits per unit time and cost (Bhat et al., 2016).

In addition to genotypic variation, modelization of phenotype should ideally include biochemical traits such as metabolomics data (Kumar et al., 2017). Systems biology approaches can help genomics studies through systematic integration of –omics data related to a particular phenotype (Yang et al., 2016), paving the way to decipher the complexity of quantitative traits by assuming models that are biologically more explicit, while retaining their predictive qualities and operability (Kadarmideen, 2014).

#### Grapevine

*Vitis vinifera* L. subsp. *silvestris* and *V. vinifera* L. subsp. *vinifera* are the main germplasm for grapevine breeding and genetic studies. QTL studies are generally developed from sexual crosses between two heterozygous parents. GWAS and GS studies take advantage of the rapid LD decay in this germplasm.

Grapevine has a diploid genome (*n* = 19) and an estimated genome size of 500 Mbp. Simple Sequence Repeat (SSR) markers from genomic and Expressed Sequence Tags (EST) have been used to determine parent-progeny relationships, to develop databases of DNA profiles for cultivar identification and for genetic mapping (Bowers et al., 1999; Adam-Blondon et al., 2004; Huang et al., 2010). More recently, numerous SNP markers were developed leading to the generation of SNP arrays for high throughput genotyping (Cabezas et al., 2011; Laucou et al., 2018). Several genetic maps (Adam-Blondon et al., 2004; Troggio et al., 2007; Wang J. et al., 2017) have been well established for *V. vinifera*, which allowed the International Grape Genome Program (IGGP) to recommend using numbering of linkage groups according to the map established by Adam-Blondon et al. (2004). A high resolution map was established from GBS data using a heterozygous mapping strategy (HetMappS; Hyma et al., 2015). Genetic maps of *Vitis amurensis* (Blasi et al., 2011), *V. rupestris*, *Vitis arizonica* (Doucleff et al., 2004) and *Muscadina rotundifolia* (Blanc et al., 2012) have been also established. Physical maps have been implemented (Scalabrin et al., 2010) and a high-quality draft of the genome sequence of grapevine (*V. vinifera*) obtained from a highly homozygous genotype was released (Jaillon et al., 2007) and further improved by (Canaguier et al., 2017). A gene nomenclature system (Grimplet et al., 2014) was implemented and a public grape genome browser^2^. Using these available tools, sequence polymorphisms and structural variations among four Sardinian grapevine cultivars have been analyzed (Mercenaro et al., 2017) and the contribution of ancestral wide species to grape breeding evaluated (Migicovsky et al., 2016).

In grapevine, QTL analysis is much more developed than in citrus or olive. GWAS analyses have also been already performed for quality traits (Laucou et al., 2018) and early ripening (Xu et al., 2016). Studies concerning all aspects of its cultivation including disease resistance (Blasi et al., 2011; Rex et al., 2014; Pap et al., 2016), quality traits in berries (Cabezas et al., 2006; Chen et al., 2015; Yang et al., 2016); growth and reproductive traits (Houel et al., 2015; Zhao et al., 2016), yield and agronomic traits such as adaptation to water deficit and lime induced iron chlorosis (Viana et al., 2013; Coupel-Ledru et al., 2014) and, particularly, the adaptation of grapevine varieties to a climate change scenario (Duchene et al., 2012), have been carried out. To this respect, GWAS studies along with the analysis of population structures have shown that LD in the domesticated grapevine is low, even at short ranges, but persists above background levels, set around 3 kb (Myles et al., 2010; Nicolas et al., 2016). Such rapid decay of LD was also observed in *Vitis vinifera* L. subsp. *silvestris* decreasing to 0.1 within 2.7 cM for genotypic data and within 1.4 cM for haplotypic data (Barnaud et al., 2010). Considering this rapid LD decay, Myles et al. (2010) suggested that for grapevine “[…] whole-genome sequencing will become the genotyping method of choice for genome-wide genetic mapping studies.”

#### Citrus

Most of the citrus germplasm results from interspecific hybridization and is highly heterozygous (Wu et al., 2014, 2018; Curk et al., 2016). Therefore, genetic mapping studies and QTL analysis are mainly based in a two-way pseudo-test cross-mapping strategy, from controlled hybridization between two heterozygous parents, and producing genetic maps for each parent (Ollitrault and Navarro, 2012). Genetic mapping and QTL studies are focused in *Citrus* and *Poncirus* species. Due to the high structuration of the citrus gene pool, composite populations built from germplasm accessions and hybrids between several parents are used for GWAS studies and the elaboration of GS models (Minamikawa et al., 2017; Imai et al., 2018).

Citrus, with a basic chromosome number of 9, has a relatively small genome size. It varies among ancestral taxa from 398 to 360 Mb/haploid genome for *C. medica* and *C. reticulata*, respectively. *C. maxima* had an intermediate genome size of 383 Mb (Ollitrault et al., 1994) and the haploid genome of *C. sinensis* was estimated to be 372 Mb. Several types of co-dominant nuclear markers have been developed for genetic studies in citrus; among the most currently used, SSR derived from genomic (Ollitrault et al., 2010; Liu et al., 2013) and from transcriptomic data (Luro et al., 2008; Liu et al., 2013; Liang et al., 2015) are found. With the development of NGS technologies, SNPs derived from genomic or transcriptomic studies have become one of the most important resources for molecular marker development (Ollitrault et al., 2012; Chen and Gmitter, 2013; Curk et al., 2015). Efficient SNPs genotyping methods have been developed for scalable experiments using competitive allele amplification [KASPar Technology; (Cuenca et al., 2013; Garcia-Lor et al., 2013)], or Cleaved Amplified Polymorphic Sequences (CAPS, Shimada et al., 2014; Omura and Shimada, 2016). Moreover, SNPs arrays have been developed for high-throughput studies (Ollitrault et al., 2012; Fujii et al., 2013). Recently, in California, a NIFA funded project^3^ re-sequenced 30 citrus species^4^ and developed Affymetrix Axion SNP arrays with about 1.556,000 SNPs. Additionally, GBS and Restriction site-Associated DNA markers sequencing or RADseq analyses have already been successfully developed in citrus (Penjor et al., 2014; Guo et al., 2015; Oueslati et al., 2017). The first reference genetic map based on co-dominant markers (SSRs, Indels and SNPs) with known flanking sequences was established for *C. clementina* (Ollitrault et al., 2012) and, subsequently, saturated maps of sweet orange (Xu et al., 2013), mandarin (Shimada et al., 2014) and pummelo (Guo et al., 2015) where published. Important success has been accomplished in the field of structural genomics in the last 10 years: reference genomes assembled in pseudomolecules were released for sweet orange (Xu et al., 2013), clementine (Wu et al., 2014) and pummelo (Wang X. et al., 2017). Most of the *Citrus* species were re-sequenced revealing the interspecific admixture of modern varieties (Wu et al., 2014, 2018). QTLs for resistances to diseases and pests (e.g., CTV, *Alternaria alternata*, nematodes and leaf miner) (Ling et al., 2000; Asins et al., 2004; Bernet et al., 2005; Cuenca et al., 2016) have been developed, and also for morphological and/or quality traits [fruit acidity, polyembryony, and apomixes; (Fang et al., 1997; Asins et al., 2015)]. Recently, GWAS studies were performed for quality traits (Curtolo et al., 2017; Minamikawa et al., 2017). Gois et al. (2016), providing evidences of the potential of genome wide selection in citrus for fruit acid and sugar contents. However, the learning and applications populations were limited to one parental combination. Working with composite populations associating traditional germplasms and recent hybrids, Minamikawa et al. (2017) and Imai et al. (2018) developed GS models for quality traits based on wider learning diversity.

Unfortunately, QTL and bulk segregant analyses for the identification of markers linked with tolerance to abiotic stresses still remain limited (Weber et al., 2003; Ben-Hayyim and Moore, 2007; Raga et al., 2016).

#### Olive

*Olea europaea* subsp. *europaea* constitute the major germplasm for olive breeding and therefore for genetic and genomic studies. Genetic maps and QTL analysis are generally developed from sexual crosses between two heterozygous varieties. The Gene Pool Method [GPM; Rugini et al., 2016)] developed for olive tree breeding is a promising tool to develop further GWAS and GS projects.

*Olea europaea* is considered a diploid species with *n* = 23 chromosomes. However, cytogenetic studies (Breviglieri and Battaglia, 1954) proposed that the species had originated from interspecific crosses, probably by parentals whose haploid chromosome numbers were *n* = 11 and *n* = 12 as occurs with several species of the Oleaceae family. The nuclear DNA content of *O. europaea* cultivars estimated by flow cytometry ranged between 1.45 and 1.53 Gb/haploid genome and the genome size of wild olive was estimated 1.59 Gb/haploid genome (Loureiro et al., 2007). Many molecular studies in olive, including the most recent, are based on dominant markers such as Random Amplified Polymorphic DNA (RAPD), Amplified Fragment Length Polymorphism (AFLP), or Inter Simple Sequence Repeats (ISSR) despite much more useful co-dominant markers have been developed during the recent years (see Sebastiani and Busconi, 2017 for review). First olive SSR markers were published by Cipriani et al. (2002) and more recently numerous SSRs have been developed from EST data (De la Rosa et al., 2013; Essalouh et al., 2014; Mariotti et al., 2016). SNP mining and marker implementation is ongoing (Reale et al., 2006; Kaya et al., 2013) and Cleaved Amplified Polymorphic Sequences (CAPS) markers have been successfully developed (Bazakos et al., 2012). Until now, there is no SNP microarray for high-throughput genotyping but GBS has been successfully developed rendering saturated maps with more than 4,000 SNP markers constituting 23 linkage groups (İpek et al., 2016). Genetic maps were published by Khadari et al. (2010) comprising mainly dominant/anonymous markers. Sadok et al. (2013) and Essalouh et al. (2014) added new markers and particularly SSRs in the map of Olivière × Arbequina olive cultivars (Khadari et al., 2010) producing the most saturated olive map generated with traditional molecular markers. Several whole genome sequencing projects engaged during the last years: one by an Italian consortium within the framework of the OLEA project (Muleo et al., 2012), the International Olive Genome Sequencing Consortium (IOGC) (Unver et al., 2017) and another IOGC project that sequenced a 1,000-years old tree of the Spanish ‘Farga’ variety (Cruz et al., 2016). Assembly provided a draft genome of 1.31 Gb, representing 95% of the estimated genome length. Nevertheless, few QTL analyses have been published so far: Sadok et al. (2013) identified 12 QTLs associated to flowering, fruiting and production whereas (Atienza et al., 2014) performed an association study with olive oil quality criteria. A genetic association study was performed with SSRs markers to develop MAS for fatty acid contents Ipek et al. (2015) and Kaya et al. (2016) reported the first GWAS study for yield-related traits.

### Polyploidization as a Feasible Approach to Enhance Tolerance to Environmental Constraints

Polyploidy has been identified as a major force driving plant evolution in order to better adapt to environmental constraints (Soltis and Soltis, 2009; Chen, 2010). Polyploid species are common in harsh environments (Brochmann et al., 2004) and investigations during the last decade showed that inducing polyploidization in diploid genomes improves stress tolerance in different woody plant species (Allario et al., 2012; Meng et al., 2016; Ruiz et al., 2016b; Greer et al., 2018). However, investigation on the role of polyploidization in stress tolerance is hampered in part by the phylogenomic history of the given plant species, the biology and the particular genetic structure (Jackson and Chen, 2010). To this respect, citrus have the advantage that, although most cultivated genotypes are diploid, apomictic seeds produced in several citrus species exhibit the natural occurrence of polyploids (Aleza et al., 2011). This has boosted the selection and investigation of polyploids in citrus as a feasible alternative in breeding.

In citrus, polyploidy is often associated to a wide range of morphological and physiological changes that are often advantageous under adverse environmental conditions (Allario et al., 2012; Ruiz et al., 2016a). For instance, citrus tetraploids are shorter and have bigger leaf area (Allario et al., 2011), have thicker mesophyll cells that have been associated to an increase in internal diffusive resistances to CO_2_, thus reducing net photosynthetic rate (Romero-Aranda et al., 1997), contributing to explain in part the dwarf phenotype observed in tetraploids (Allario et al., 2011).

Spontaneous doubled-diploid genotypes (chromosome set duplication) occur in seedlings of apomictic citrus (Aleza et al., 2011). They show several interesting features that can be exploited in plant breeding for abiotic stress tolerance. For instance, when subjected to drought, they show better acclimation traits than the respective diploid linked to a more limited transpiration and the maintenance of higher leaf water content (Oliveira et al., 2017), while a specific root architecture with reduced ramification, shorter root diameter with higher specific length at the morphological level has been linked to increased radial hydraulic conductivities (Lp_r_). This influences whole plant water status as well as plant growth and development, contributing to the enhanced tolerance to water deprivation (Ruiz et al., 2016a,b). Moreover, tetraploid leaves show fewer and larger stomata compared to 2*n* which has been associated to decreased stomatal conductance and transpiration (Allario et al., 2011; Padoan et al., 2013). Using polyploid Rangpur lime as rootstock, (Allario et al., 2012) improved performance of diploid *C. sinensis* cv. Valencia Delta under water stress. This response was linked to a basal constitutive up regulation of genes involved in compatible osmolyte and hormone biosynthesis (Allario et al., 2012) and detoxification of ROS (Tan et al., 2015).

These interesting behaviors of tetraploid citrus against abiotic stresses result in an increasing interest for tetraploid rootstock breeding. New tetraploid rootstocks are obtained by spontaneous occurrence of doubled-diploid embryos from apomictic seeds (Aleza et al., 2011), by somatic hybridization (Dambier et al., 2011) or sexual hybridization between complementary tetraploid rootstock (tetrazyg strategy; Grosser et al., 2015).

Despite being less common, polyploidy in grapevine and olive constitutes an interesting strategy to improve reproductive and yield traits.

In grapevine, for instance, autotetraploid rootstocks reduce the vigor of the scion compared with their parental diploid while maintaining Phylloxera resistance (Motosugi et al., 2002). Polyploidy also increased berry size as well as concentration of sensory and bioactive compounds (Acanda et al., 2015); moreover, the generation of triploids is also particularly interesting for the production of seedless table grapes.

A few natural tetraploid olive trees have been identified in the *O. europea* complex (Besnard et al., 2007) or isolated from mixoploid mutants (Rugini et al., 1996) showing a significant reduction of trunk and canopy size (Rugini et al., 2016). The natural occurrence of polyploids in olive has been confirmed for different populations and subspecies, being triploids less frequent than tetraploid (Sebastiani and Busconi, 2017). It has been shown that tetraploidy induces changes in floral and fruit morphology, producing larger floral structures and increasing the pistil abortion rate compared to diploids, but surprisingly, no significant effect on fruit size could be observed (Caporali et al., 2014). Unfortunately, no studies associating polyploidization with abiotic stress tolerance are currently available in grapevine or olive, although evidence from citrus and other experimental systems points toward a positive effect.

## Systems Biology Tools to Integrate Physiological, Biochemical and Molecular Data to Build Predictive Models

The ability of plants to withstand adverse environmental conditions and survive is dependent upon the activation of highly coordinated protective responses (Peleg and Blumwald, 2011), resulting from the interaction between specific genetic components, the genetic landscape as well as with the particular environmental factors. This interaction is highly complex, therefore, the translation of genetic responses to field performance is not straightforward and many different factors may influence the outcome.

Systems biology approaches study biological systems from a multidisciplinary point of view aided by mathematical modeling. To this respect, interaction network analysis constitutes a data-driven approach that represents a biological component (e.g., a gene or a protein) as a node and its physical, genetic and/or functional interactions as edges that help to visualize and interpret multivariate datasets. These representations allow identification of functional modules, transcriptional circuits or signaling pathways contributing to characterize a biological system (Ideker and Krogan, 2012). This approach can be applied not only to investigate the participation of different signaling or metabolic pathways in the response to stress but also to infer novel interactions among different network nodes and to predict unknown gene functions (Avin-wittenberg et al., 2012; Rhee and Mutwil, 2014).

The power of network analysis to reveal novel interactions relies largely on the use and integration of several –omics technologies (Fukushima and Kusano, 2014). These techniques provide an unbiased and non-targeted analysis of macromolecules and small molecular weight metabolites. To this respect, transcriptomics and metabolomics data can be combined to construct a network that displays the degree of association between genes and metabolites and depicts levels of co-expression among molecules. Nevertheless, it is important to note that network analysis only provides a reliable hypothesis since correlation does not necessarily implies causal relationship between the different components. Therefore, protein–protein (PPI), protein–DNA interaction studies as well as direct/reverse genetics approaches are required to confirm those interactions. Several online tools for network construction and analysis using co-expression data are available (Table 1).

**Table 1:** Main grapevine, citrus and olive species already sequenced including working URLs with access to JBrowse, BLAST search and multiple sequence alignment tools and availability of systems biology tools with currently workingURLs.

Plant Species	Estimated genome size	Genomic resources	Systems biology resources
***Vitis vinifera* L.**	500 Mbp (Jaillon et al., 2007)	Accessible from:	• Vitisnet (https://www.sdstate.edu/agronomy-horticulture-and-plant-science/functional-genomics-bud-endodormancy-induction-grapevines-5) genome sequences and ESTs from the *Vitis* genus, spans more than 39,000 unique sequences and 13,145 genes assigned to 219 networks (Grimplet et al., 2009).
		www.genoscope.cns.fr/externe/GenomeBrowser/Vitis	• VTCdb based on the publicly available microarray data from *Vitis vinifera* Affymetrix 16K GeneChip and the NimbleGen Grape Whole-genome microarray chip (29K), spans over 29,000 genes (Wong et al., 2013) **no longer available.**
		https://phytozome.jgi.doe.gov/pz/portal.html#!info?alias=Org_Vvinifera	• Biowine (https://alpha.dmi.unict.it/biowine/) designed for the functional analysis of genomes of Sicilian grapevine cultivars. This system allows the analysis of RNA-Seq including the connection to miRNAs (Pulvirenti et al., 2015).
		http://genomes.cribi.unipd.it/grape/index.php or GenBank taxa id 29760	• miRVIT (http://mirvit.ipsp.cnr.it/) updated miRNA accession list repositioned according to the most recent *V. vinifera* genome (Chitarra et al., 2018).
			• VESPUCCI (http://vespucci.colombos.fmach.it/) comprises most microarray and RNA-Seq data for *V. vinifera* (Moretto et al., 2016).
***Citrus sinensis* L. Osbeck**	367 Mbp (Xu et al., 2013)	Accessible from: https://phytozome.jgi.doe.gov/pz/portal.html#!info?alias=Org_Csinensis or GenBank taxa id: 2711	**None available**
***Citrus clementina* L.**	302 Mbp (Wu et al., 2014)	Accessible from: https://phytozome.jgi.doe.gov/pz/portal.html#!info?alias=Org_Cclementina or GenBank taxa id: 85681	
***Citrus reticulata* L.**	370 Mbp (Wang L. et al., 2018)	Accessible from: https://www.citrusgenomedb.org/organism/5941 or GenBank taxa id: 85571	
***Citrus medica Citrus maxima Citrus ichangensis***	407 Mbp, 380 Mbp, 391 Mbp (Wang X. et al., 2017; Wu et al., 2018)	Accessible from: *C. medica* https://www.citrusgenomedb.org/organism/5945 or GenBank taxa id: 171251 *C. maxima* https://www.citrusgenomedb.org/organism/5947 or GenBank taxa id: 37334 *C. ichangensis* https://www.citrusgenomedb.org/organism/5948 or GenBank taxa id: 2709	
***Citrus unshiu* L.**	370 Mbp (Shimizu et al., 2017)	Accessible from: http://www.citrusgenome.jp/ or GenBank taxa id: 55188	
***Olea europaea* L**	1380 Mbp (Cruz et al., 2016; Unver et al., 2017)	Accessible from subsp. europeae GenBank taxa id: 4146 subsp. sylvestris http://olivegenome.org/ or GenBank taxa id: 158386	• Olive Genetic Diversity Database (OGDD) (http://www.bioinfo-cbs.org/ogdd/), a genetic, morphologic and chemical database of worldwide olive oil production for the identification of unknown olive tree cultivars, based on SSR markers (Ben Ayed et al., 2016).


Several authors have highlighted the importance of implementing systems biology approaches for unraveling the mechanisms responsible for plant desiccation tolerance and how these are intertwined, especially when this knowledge has to be transferred to cultivated species and when genetic engineering strategies for improving crop tolerance to drought are involved (Moore et al., 2009 and references therein).

### Grapevine

Since the release of *V. vinifera* genome in 2007 (Jaillon et al., 2007), several efforts have been made toward the identification and functional annotation of all gene-encoding sequences (Grimplet et al., 2012) including the genome-wide analysis of all potential *cis*-regulatory elements (Wong et al., 2017), miRNA-encoding sequences and their potential target genes (Jiu et al., 2015) and the specific study of several gene families associated to stress responses such as dehydrins(Yang et al., 2012), CBL, CIPKs (Xi et al., 2017), and CDPKs (Zhang et al., 2015) or involved in developmental processes such as MADs-box transcription factors (Grimplet et al., 2016).

In the post-genomics era, future breeding strategies rely on the investigation of abiotic stress tolerance responses and their correlation to genetic traits (Cramer, 2010; Yang et al., 2012; Dal Santo et al., 2016; Fortes and Gallusci, 2017). Gene co-expression networks (GCN), based in the notion that genes involved in similar or related processes exhibit similar expression patterns under different experimental conditions, are the preferred strategy (Wong et al., 2013). To this respect, several online platforms have been developed for *V. vinifera* (Table 1).

In this new scenario, rational and precise breeding strategies require the unambiguous identification of ‘stress tolerant’ phenotypes to be associated to specific expression patterns. In a pioneering work, Cramer et al. (2007) and Deluc et al. (2007) included transcript and metabolite analyses in vegetative and reproductive tissues of grapevine, respectively, depicting changes in response to abiotic stresses and during berry development. The manual integration of transcript and metabolite data allowed the identification of RuBisCo activase as an early response to water deprivation that was delayed under saline conditions. Moreover, water stress affected metabolism-associated transcripts whereas salinity had an impact mostly on protein synthesis and fate (Cramer et al., 2007). The transcript profiling data correlated well with metabolite profiles showing water-deprived grapevine tissues higher concentrations of glucose, malate, and proline than salt-stressed ones. Unfortunately, although this work included metabolite profiling data, no attempt to correlate transcript and metabolite profiling was carried out to provide a more insightful view of the regulatory processes involved in metabolite accumulation. In a recent work, Corso et al. (2016) performed RNA-Seq on leaves and roots of different grapevine genotypes with contrasting ability to tolerate water deprivation. Transcriptome analysis revealed the WRKY transcription factors (TFs)-dependent activation of secondary metabolism potentially leading to the accumulation of stilbenes and flavonoids. However, this extent could not be confirmed with a parallel metabolite analysis and no co-expression analysis to unbiasedly correlate specific TF activity with the induction of the biochemical response was attained. In both cases, the use of integrative approaches could have contributed to extract more significant knowledge from already collected data.

Recently, Wong and Matus (2017) generated a composite network in grapevine by overlaying co-expression maps between structural and TF genes, integrated with the presence of promoter *cis*-binding elements, miRNAs, and long non-coding RNAs (lncRNA) focusing on the phenylpropanoid pathway. This allowed the characterization of novel TFs and miRNAs potentially involved in the regulation of the phenylpropanoid pathway. Following a similar procedure, but integrating metabolomics, transcriptomics and proteomics data of grapevine berries in different developmental stages, Zamboni et al. (2010) identified biomarkers of berry development and senescence.

Analysis of GCN has been applied in grapevine to identify ripening-associated functional sub-modules under water stress conditions (Savoi et al., 2017), revealing a strong interplay between key metabolites and structural genes and the involvement of VviMYBA1-2 in anthocyanin biosynthesis. In this work, GCN revealed major drought stress-regulated gene modules linked to central and specialized metabolites and multiple signal transduction pathways (e.g., anthocyanin and amino acid biosynthesis via members of VviAP2/ERF and VviNAC TF families). The activation of both abscisic acid (ABA)-dependent and -independent pathways may act balancing the regulation of the stress response and the berry ripening program.

### Citrus

Sequencing of sweet orange and the major cultivated citrus varieties and related genera including the three parental lines: pummelo, mandarin and citron (Xu et al., 2013; Wu et al., 2014; Shimizu et al., 2017; Wang X. et al., 2017) (Table 1) has played a pivotal role in the development of biotechnological tools in this woody crop. For instance, the generation of a reference RNA-Seq transcriptome revealed the existence of 3,326 new genes and increased the number of alternative splicing variants (Terol et al., 2016). Annotation of gene function in newly sequenced genomes is not a trivial aspect most efforts are oriented toward the functional annotation of all encoding sequences in the citrus genome. Nowadays, with increasing volumes of transcriptomics data available for *Citrus* species, GCN has become a viable option for predicting gene function at a genome-wide scale targeting genes encoding proteins and/or other non-protein coding RNAs. Compared with classical GO surveys, GCN poses the advantage of allowing the annotation of new genes for which no plausible ortholog in better annotated plant species exists (Wong et al., 2014).

In the pre-genomic era, matching available and annotated ESTs from the Citrus Genome Sequencing Consortium (CitEST) database (Forment et al., 2005) was the only way to attain this kind of studies (Quecini et al., 2007). Using this approach, crucial stress responses in salt-tolerant (Cleopatra mandarin) and –sensitive (Carrizo citrange) citrus rootstocks have been studied (Brumós et al., 2009), confirmed the involvement of ABA in the constitutive tolerance of tetraploid Rangpur lime (*C. limonia*) (Allario et al., 2011, 2012), the responses of a cultivated mandarin accession to water deprivation (Gimeno et al., 2009) or the involvement of gibberellins in the regulation of photosynthesis under different abiotic stress conditions (Huerta et al., 2009) studied.

As in grapevine, most integrative studies have so far focused on fruit ripening and quality. Recently, Ibáñez et al. (2014) integrated transcript and metabolite profiles to decipher the metabolic and physiological processes underlying citrus fruit rind puffing, a disorder that produces ‘swollen’ fruits that are usually rejected. A PPI analysis inferred from previous work on *Arabidopsis thaliana*, revealed glycolysis and TCA as functional modules being severely affected in the puffing disorder. Moreover, this study also suggested that cytokinins and gibberellins could act repressing the symptoms of the puffing disorder (Ibáñez et al., 2014). In another work, postharvest disease resistance of thermally acclimated vs. non-acclimated fruits was investigated through the integration of proteomic and metabolomic profiles in satsuma mandarins. Proteins annotated as glucanases, class III chitinases and a 17.7 kDa heat shock protein were up-regulated in thermally acclimated fruits whereas enzymes involved in redox metabolism were downregulated (e.g., isoflavone reductase, superoxide dismutase, etc.). Protein data correlated with increased levels of fatty acids, amino acids, carbohydrates, organic acids, and different secondary metabolites known to be involved in reducing the effects of external stress (Yun et al., 2013).

Focusing on abiotic stress, a recent work studied a co-expression network including both mRNA and non-coding miRNAs and a method to identify miRNA-transcription regulator-target connections in citrus was described (Khodadadi et al., 2017). miRNAs have been shown to play a pivotal role in the regulation of abiotic stress responses in plants (Urano et al., 2010; Ragupathy et al., 2016). The genus *Poncirus* with its only representative *P. trifoliata* L. Raf constitutes an example of cold-temperature acclimation in citrus. Hence, the study of cold acclimation in this genotype revealed the involvement of 107 conserved miRNAs and 5 potential novel miRNAs targeting stress-responsive genes (Zhang et al., 2014). A miRNA-target mRNA regulatory network was elaborated in citrus roots in response to dehydration and salinity (Xie et al., 2017). A total of 76 miRNA (47 conserved and 29 novel) were altered either by salt stress, desiccation or both. The correlation network was partially consistent regarding differential regulation of miRNA and their respective targets, although the existence of another layer of interaction involving some of the miRNA-regulated genes acting as transcriptional inducers/repressors was also proposed.

Protein interaction with other proteins and other biomolecules is crucial in every cell process and understanding how these interactions take place is the ultimate goal of systems biology. To this respect, protein sequence and post-translational modifications (PTMs) are two essential aspects to consider. In recent works, Tanou et al. (2012) and Ziogas et al. (2015) investigated the occurrence of several PTMs in sour orange plants subjected to NaCl and PEG stress with/without pre-treatment with priming agents. Results indicated that carbonylation, *S*-nitrosylation, and Tyr-nitration under stressful conditions were the most prominent PTMs also highlighting the existence of ‘universal players’ in the stress response, such as carbonic anhydrase and chlorophyll *a-b* binding protein (Ziogas et al., 2015). In this sense, S and N donors used as priming agents modified the PTMs pattern altering protein biochemical activity and binding affinity.

### Olive

Despite the agricultural and cultural heritage importance of olive tree, the state-of-the-art in -omics toolkits for systems biology in olive is considerably delayed respect to grapevine or citrus and very few reports employing systems biology approaches in this crop exist to date.

Olive trees have a considerable ability to adapt to harsh environments due to their outstanding phenotypic plasticity. Nevertheless, global climate change poses a challenging scenario for olive tree cultivation and productivity, potentially leading to significant decreases in olive fruit and oil yield (Fernández, 2014).

Until recently, genomic studies in olive focused mainly on the employment of molecular markers toward identification of genetic variability, olive oil traceability and parental progeny analysis to aid in genetic improvement programs (Sebastiani and Busconi, 2017). Nevertheless, as in citrus, pre-genomic molecular biology was still possible with different EST collections whose components were functionally annotated by means of ortholog BLAST search. In a series of experiments carried out using two *O. europaea* cultivars with contrasting salt stress tolerance, the genotype-dependent differences in the transcriptional response were evaluated (Bazakos et al., 2012). Moreover, the resulting expression data was integrated using network analysis to infer regulatory processes aiming at inducing adaptive responses. As a result, several homologs of ERF, bZIP, and NF-Y TFs families were characterized. In other plant species, ERF TFs are known to regulate ABA biosynthesis in a stress-related manner. In turn, bZIPs appeared to act as master regulators of other TFs within the gene regulatory network, seemingly acting upstream the olive tree ERF homolog. As a result of this study, it was shown that NF-Y TF is located at the uppermost hierarchical position in the regulatory network potentially regulating rewiring of node connectivity in response to salt stress. In general, the salt-tolerant genotype exhibited a more complex TF interaction network than the salt-sensitive, suggesting the existence of a more orchestrated and progressive response to salt stress. More recently, another in-depth report made by the same authors employing 454 pyrosequencing (Bazakos et al., 2015) reinforced and expanded the findings reported in the original 2012 manuscript. Notably, both transcriptomic analyses highlighted the involvement of the same members of the regulatory networks but the more recent study considerably extended the list of involved TFs including JERF, GRAS, and HMG homologs (Bazakos et al., 2015). Furthermore, a significant cluster of ABA-related unique transcripts were identified, reinforcing the significant role of ABA in salt stress responses and/or adaptation of olive trees to stressful conditions.

Most recently available studies using systems biology tools in olive have so far relied mainly on transcriptomic approaches focusing on regulatory processes during developmental events, namely flower development (Alagna et al., 2016), development of the pollen tube (Iaria et al., 2016), as well as overall plant architecture (González-Plaza et al., 2016). Others aimed to decipher protein regulatory processes during fruit development (Bianco et al., 2013), while targeted metabolite profiles were successfully mapped for important bioactive components such as polyphenols (Medina et al., 2012) and vitamin E (Georgiadou et al., 2015, 2016). Non-targeted approaches could, however, mine important information that could give significant insight into agronomic issues (Ben Ayed et al., 2016) or stress-related processes. A recent study using a non-targeted metabolomic profiling approach in different olive tree tissues revealed 5,776 metabolite-metabolite correlations and highlighted the upregulation of biosynthetic pathways for phenylpropanoids, monolactams, indole alkaloids and flavonoids especially in young leaves. Considering the well-documented involvement of phenylpropanoids, indole alkaloids and flavonoids in stress protection phenomena, a general defense mechanism was proposed suggesting that metabolites involved in the resistance to biotic and abiotic stresses are mainly biosynthesized in new leaves (Rao et al., 2017).

### Integration of –Omics for the Selection of Plant Material in Breeding Programs

The ultimate goal of plant systems biology is to fully understand how plants respond to their environment. To this respect, gene/trait expression, is a multi-scale and highly dynamic process consisting of several highly regulated steps (1) transcription, which is regulated through the interaction of TFs and *cis*-regulatory elements present in DNA as well as epigenetic changes that modify DNA accessibility, (2) RNA processing through several post-transcriptional modifications such as the removal of introns or splicing, chemical modification of nucleotides (e.g., for rRNA) or the removal of particular sequences, allowing the acquisition of specific secondary structures (e.g., miRNA), (3) translation which is also influenced by proteins such as eukaryotic initiation factors involved in ribosome assembly and miRNAs and (4) post-translational modifications (partial proteolysis, phosphorylation, sumoylation, etc.) that affect protein activity. What is more, multidimensional interactions involving proteins, protein-nucleic acids and also different metabolites (regulatory compounds such as plant hormones, allosteric modifiers, etc.) need to be added. In such a complex regulatory scenario, the integration of –omics within network analysis provides a data-driven and dynamic scaffold to select relevant genes involved in the regulation and development of the phenotype.

This extent has been so far poorly investigated in woody crops despite similar approaches proved to be successful in identifying relevant genes in other plant species. In a pioneering study, Morreel et al. (2006) combined metabolite profiling and QTL analyses to identify loci controlling metabolite abundances in a full-sib poplar population. Metabolite correlation networks indicated a tight association of biosynthesis of specific flavones/dihydroflavonols and flavonols. More recently, integration of metabolomics and transcriptomics in co-expression analyses contributed to identify genes involved in side-chain elongation steps of aliphatic glucosinolate biosynthesis (Albinsky et al., 2010). In woody crops, similar approaches have been applied to the identification of genes controlling acidity in oranges (Huang et al., 2016), revealing the importance of proteins involved in metabolite transport and degradation acting as hubs in the citrate accumulation gene network. In grapevine, integration of metabolomics and transcriptomics revealed the involvement of typical abiotic stress-responsive transcription factors such as bZIPs, AP2/ERFs, MYBs and NACs in the accumulation of metabolites relevant to grape berry quality under unfavorable conditions (Savoi et al., 2017). Also in grapevine, integration of transcriptomics and proteomics allowed the identification of transcriptional, post-transcriptional and translational mechanisms involved in the responses of grapevine to high temperatures. This study revealed that alternative splicing constitutes an important mechanism in the response of grapevine to climate change conditions (Jiang et al., 2017). Overall, all evidence points out that systems biology approaches are useful data-driven strategies to identify relevant genes involved in different plant processes.

## Future Prospects

The constraints to global food production posed by climate change conditions have become a key issue in agronomy and plant physiology. Perennial plant species, such as the ones in which this review focuses, are subjected to adverse environmental conditions throughout their entire life cycle, and over consecutive cropping seasons. Therefore, breeding of new varieties more resilient to abiotic stresses with improved performance and productivity traits constitutes a prioritary objective. Efficient plant breeding requires accurate phenotyping of plant traits reliably associated to stress tolerance, enhanced productivity and/or quality. The development of high-throughput phenotyping platforms complements fruit tree genomics bridging phenotype and genotype, allowing GWAS and network analyses expanding our knowledge on genetic responses linked to the development of particular phenotype traits. In the field of abiotic stress tolerance, as an example, the integration of -omics for the engineering and selection of abiotic stress-tolerant genotypes has been successfully applied to staple and forage crops (Deshmukh et al., 2014; Singh et al., 2015; Shah et al., 2018). Nevertheless, despite the existing technological gap between fruit trees, staple and forage crops and model plants in terms of genomic resources, databases and tools, the situation is changing rapidly and the amount of sequenced genomes and gene association studies increasing. It is therefore desirable that, in the following years, curated databases appear including data from different sources and technological platforms. Abiotic stress tolerance traits are complex from the genetic point of view involving an intricate interaction of regulator and effector genes. Therefore, the systems-level analysis of complex phenotype traits requires a complete evaluation of the relevant physiological parameters to integrate the genetic and molecular differences, constituting the real ‘driving force’ in the selection process. Therefore, the integration of accurate phenotype data along with gene expression, metabolite and protein accumulation in different tissues and germplasm accessions would allow researchers performing *in silico* gene association studies, as in *A. thaliana*, and select candidate genes for genetic improvement. This will undeniably boost any woody crop breeding initiative by narrowing down the number of target gene(s) and providing a clear gene-to-phenotype association.

## Author Contributions

CDO and VA conceived and drafted first manuscript organization. CDO, RM, VF, JP, PO, AG-C, and VA wrote the manuscript and produced tables and figures. All authors revised subsequent drafts of the manuscript and approved final version.

## Conflict of Interest Statement

The authors declare that the research was conducted in the absence of any commercial or financial relationships that could be construed as a potential conflict of interest.

## Footnotes

1 http://www.oiv.int

2 www.genoscope.cns.fr/externe/GenomeBrowser/Vitis

3 https://portal.nifa.usda.gov/web/crisprojectpages/1001031-development-and-application-of-a-high-density-snp-genotyping-array-for-citrus.html

4 https://www.citrusgenomedb.org/analysis/185
